# Eliminating the high abortion related complications and deaths in Cameroon: the restrictive legal atmosphere on abortions is no acceptable excuse

**DOI:** 10.1186/s12905-018-0564-6

**Published:** 2018-05-24

**Authors:** Luchuo Engelbert Bain, Eugene Justine Kongnyuy

**Affiliations:** 1 Centre for Population Studies and Health Promotion, CPSHP, Yaounde, Cameroon; 2 Athena Institute for Research on Innovation and Communication in Health & Life Sciences, Faculty of Earth and Life Sciences, Vrije Universiteit, Amsterdam, The Netherlands; 3 United Nations Population Fund (UNFPA), Abuja, Nigeria

**Keywords:** Abortions, Complications, Deaths, Cameroon, Restrictive, Legal atmosphere

## Abstract

**Background:**

The abortion law in Cameroon is highly restrictive. The law permits induced abortions only when the woman’s life is at risk, to preserve her physical and mental health, and on grounds of rape or incest. Unsafe abortions remain rampant with however rare reported cases of persecution, even when these abortions are proven to have been carried out illegally.

**Discussion:**

Available public health interventions are cheap and feasible (Misoprostol and Manual Vacuum Aspiration in post abortion care, modern contraception, post-abortion counseling), and must be implemented to reduce unacceptably high maternal mortality rates in the country which still stand at as high as 596/100.000. Changes in the legal status of abortions might take a long time to come by. Albeit, advocacy efforts must be reinforced to render the law more liberal to permit women to seek safe abortion services. The frequency of abortions, generally clandestine, in this restrictive legal atmosphere has adverse economic, health and social justice implications.

**Conclusion:**

We argue that a non-optimal or restrictive legal atmosphere is not an acceptable excuse to justify these high maternal deaths resulting from unsafe abortions, especially in Cameroon where unsafe abortions remain rampant. Implementing currently available, cheap and effective evidence based practice guidelines are possible in the country. Expansion and use of Manual Vacuum Aspiration kits in health care facilities, post-abortion misoprostol and carefully considering the content of post abortion counseling packages deserve keen attention. More large scale qualitative and quantitative studies nationwide to identify and act on context specific barriers to contraception use and abortion related stigma are urgently needed.

## Background

The maternal mortality rate in Cameroon as of 2015 was estimated at 596/100.000, a remarkable but far from enviable decrease from 728/100.000 in 1990 [1]. Mortality rates as high as 1266/ 100.000 have been reported in the Far North Region of Cameroon [2]. Over 20 million unsafe abortions are carried out each year with 47,000 of these women ending up dying worldwide [3]. Most of these deaths do occur in developing countries. Despite the overall decline in abortion rates worldwide, the prevalence has remained constant in developing countries [4]. Over thirteen (13%) of maternal deaths are as a result of unsafe abortions [3]. Despite the restrictive abortion laws in Cameroon, induced abortion rates remain high. Mosoko et al. reported over 35% of females aged 24 or more attending 6 antenatal clinics in the political headquarters in Yaounde having carried out an abortion in the past [5]. Calvès also reported that 35% of pregnancies amongst adolescents and early adulthood do end up in abortions [6]. Adonis et al. reported 19% of teenage mothers reporting having carried between 1 to 4 abortions in the past [7]. It becomes clear that despite the fact that abortion practice remains restricted, it is not uncommon [4, 7–9]. Under the present restrictive law, it is reasonable to suspect that most of these could be done under unsafe conditions. Reported methods used to induce these unsafe abortions range from transcervical foreign bodies, injections, unspecified medications and diverse plants [10, 11]. Women who survive could suffer from infections, uterine and bowel perforations, infertility, sepsis, conditions generally costly to patients and the health system. Tebeu et al. have reported over 25% of maternal deaths in a reference teaching hospital in Cameroon resulted from complications of unsafe abortions [12].

## Abortions and family planning services

Abortions are not rare in Cameroon. One fifth of Cameroonian teenage mothers have had at least one induced abortion in the past [7]. Forty percent (40%) of women aged 20 and above report at least two abortions [9]. A quarter of in hospital maternal deaths result from unsafe abortions [2]. Meeting family planning goals alone can cut down the number of abortion related maternal deaths by a third [3]. In Cameroon, the modern contraceptive prevalence is reported at 16% while the total fertility rate is 5.1 children per woman [13]. Twenty one (21%) of adolescent school girls in Cameroon are sexually active [14]. Use of contraception services, especially amongst adolescents in Cameroon is sub- optimal [15–19]. Over 40% of pregnancies in Cameroon are unplanned and 63% of women wishing to avoid a pregnancy do not use a modern method of contraception [20]. Seventy two (72%) of sexually active youths do not use condoms consistently during [17]. Between 9 to 13% of all pregnancies in Cameroon are teenage pregnancies [21, 22]. Pregnancy outcomes amongst this age group generally carry a poorer prognosis [7, 12, 21, 22]. Modern family planning services must constitute a priority action area. Not only shall this present a unique opportunity to prevent unplanned pregnancies and consequent abortion related deaths, reduction in HIV transmission with increased condom use could be parallel outcome. In Cameroon, like most African settings, discussing issues related to sexuality is generally considered a taboo. Sex education packages need to be carefully evaluated and imperatively incorporated into the secondary school education system. Kongnyuy et al. have described proper family planning services as an opportunity to dispel wrong perspectives. For instance, some women believe hormonal contraception is protective against HIV [18]. Partner involvement in decision making has been reported to influence uptake and adherence family planning methods options [15]. Elsewhere, the male partners have been reported to be highly influential in the decision making process when it comes to seeking abortion services [23]. Meeting half of the unmet contraceptive needs of Cameroon women would avoid 600 maternal deaths annually, as well as avert 65,000 unsafe abortions [20]. The World Health Organization (WHO) has described unsafe abortion related deaths as easily preventable, especially through correct use of modern contraception [3]. To be successful in eliminating preventable maternal deaths, the Maternal Death Surveillance and Response (MDSR) strategy, as described by WHO, could be a useful strategy in supporting in the identification of key drivers, and the conditions under which these unsafe abortions do take place [24, 25]. Large scale nationwide qualitative and quantitative studies to properly ascertain key barriers to modern contraception uptake are highly needed.

## Abortion care and the law

The law in Cameroon permits induced abortions only when the woman’s life is at risk, to preserve her physical and mental health, and on grounds of rape or incest [26]. Even under these clauses, obtaining a legal abortion remains cumbersome. For instance, the law states that [26]:

*“The doctor shall obtain the opinion of two experts each chosen respectively from legal experts and members of the National Council of Medical Practitioners. The latter shall testify in writing that the life of the mother can only be safeguarded by means of the intervention. The protocol of consultation shall be made in 3 copies one of which shall be handed to the patient and the other two to the consultant physician and legal expert. Besides, a protocol of the decision taken shall be sent by registered mail to the chairperson of the National Council of Medical Practitioners.”*

This law might be considered problematic on two fronts: firstly, in constituting the legally recommended team, and secondly, most women eligible for legally accepted abortions might not even be aware of the law. With a scarcity of physicians in health facilities, not to talk of specialists, obtaining legal abortions on request becomes an illusion [27]. In order words, the legal framing of the law could push women to inevitably seek abortions elsewhere (unsafe). As a signatory to the Maputo protocol (African Charter on Human and People’s Rights on the Rights of Women in Africa), legal provisions towards abortion on request should be feasible, realistic and policies surrounding the practice geared towards respecting the reproductive rights of women.

Elsewhere, section 337 of the Cameroonian penal code regarding abortions reads as follows [28]:

*“(1) Any woman procuring or consenting to her own abortion shall be punished with imprisonment for from fifteen days to one year or with fine of from five thousand to two hundred thousand francs or with both such imprisonment and fine.”*

*(2) Whoever procures the abortion of a woman, notwithstanding her consent, shall be punished with imprisonment for from one to five years and with fine of from one hundred thousand to two million francs.*

*(3) The penalties prescribed by subsection (2) shall be doubled where the offender –*

*(a) Engages habitually in abortion; or*

*(b) Practises the profession of medicine or an allied profession.*

*(4) “In the circumstances of subsection (3) (b), the Court may also order closure of the professional premises and impose a ban on his occupation under sections 34 and 36 of this Code.”*

The wave of change in most African countries regarding the ratification and gradual adoption of more liberal laws regarding abortions has left Cameroon indifferent [29–31]. Authoritative bodies like the Society of Gynecologists and Obstetricians of Cameroon (SOGOC), The National Bar Association, and the Cameroon Medical Council have been relatively silent in pushing towards adoption of more liberal laws. In Ethiopia for instance, the role of the Ethiopian Society of Obstetricians & Gynecologists (ESOG) was fundamental in rendering the law on abortion in the country more liberal in 2005 [32]. It is possibly time for these organizations to speak up. The Catholic Church for instance is staunchly against any form of abortion. Forcing women to bear children with legal tenets, to die obtaining unsafe abortions, to seek for abortion services late in the pregnancy course, or spend rare resources in managing complications resulting from unsafe abortions is not only irrational from the economic point of view, but also constitutes an unacceptable threat to the very reproductive autonomy of the woman.

Bearing children issued from rape, or other forms of sexual abuse might have far reaching negative implications for the mother and the baby (social bonding, dropping out of school, limiting socioeconomic potential of the girl child, sub – optimal upbringing of the child). Some women are forced to engage in prostitution to raise income in raising the child [27]. The double burden of having to manage the psychological trauma from rape or sexual violence, as well as exposing oneself to prostitution in order to raise the child might not be very rare, especially amongst persons of the lower socioeconomic class.

Unsafe abortions remain rampant with rare reported cases of persecution even when these abortions are carried out illegally. Women meeting conditions for a legal abortion [33] might not even know they are eligible (e.g rape, fetal malformations, and medical reasons). A restrictive legal atmosphere does not prevent women from obtaining unsafe abortions. There is compelling evidence which indicates that liberal abortion laws are associated with fewer unsafe abortions and abortion related complications and deaths [4, 8, 34]. The law on its own however, even in settings considered most liberal, does not always guarantee access to safe abortion services [33]. Even in settings with liberal abortion laws, women’s knowledge with regards to the law remains sub – optimal [33, 35]. Irrespective of the restrictive law reality, we cannot continue to observe thousands of women die every year from an easily preventable cause like unsafe abortions. In a qualitative study with 65 in depth interviewees who sought for abortion services in the Cameroon, women would prefer to keep abortions secret [23]. With most of these carried out under unsafe circumstances, easily preventable maternal deaths could be recorded since competent health care providers could be approached either late during the complication phase, or at times never. The laws disproportionately affect women of the lower socioeconomic class more [36]. Richer women can always approach private clinic staff to pay for induced abortions, while the poor are left to the mercy of clandestine abortion providers. The scope of choice for the poor is not only reduced, but also channeled in a direction with high possible adverse outcomes. A liberal legal framework in the abortion discourse not only enhances female reproductive autonomy, but also resolves a key social justice concern within the scope of reproductive health.

## Elimination of unsafe abortion related complications and deaths in Cameroon

Post Abortion Care (PAC) services are provided in Cameroon on a case by case basis. A national policy is difficult to be established and implemented due to the illegality of abortion provision, as well as the absence of a Universal Health Insurance Scheme. Poorer women disproportionately seek for clandestine abortion providers, who do not generally have the expertise and competence to manage possible complications. However, only women obtaining induced abortions from health care facilities could benefit from these. Post abortion counseling is a unique opportunity to insist on potential and adapted methods of contraception. Health care workers should have as a mandate, contraception counseling before discharging women at post partum or who have had an induced abortion.

Equipping facilities with Manual Vacuum Aspirators should be expanded throughout the national territory. Training both doctors, midwives and nurses on the technique, and making the equipment always available could help curb deaths that could result from incomplete abortions [37]. While nurses/midwives are allowed to undertake Manual Vacuum Aspiration (MVA) in Cameroon, access to MVA is limited due to lack of training and MVA kits [37]. Misoprostol is increasingly used in post abortion care. Not only is it effective in preventing abortion related complications like bleeding and infection, it is acceptable for clients and health care providers, cheap, cost – effective and associated with less abortion related stigma [38]. Osur et al. (2013) have reported a positive experience with use of Misprostol in post abortion care in Uganda and Kenya. Their findings are supportive of the cost – effectiveness, ease in scaling up especially in regions of the world with high abortion related mortality and morbidity [38]. Tumasang et al. (2014) in the Centre Region of Cameroon (Yaounde) have reported a dramatic increase in MVA use in health facilities where they were provided kits for a pilot project. They however reported unavailability of these kits during the nights. Scaling up of Misoprostol and MVA services in health facilities is not only highly needed, but appears feasible [37, 38].

The demand for abortion services remains an unavoidable reality. Policies to reduce the demand of induced abortions are to be encouraged, as well as those to render this service safer when the need arises. Improved access to, and putting in place of interventions to increase modern contraception uptake remain a major action area. The sad reality is that unintended pregnancies cannot be completely eradicated even with improved contraception uptake. Optimal contraceptive use will certainly reduce the demand for safe abortion services. Developing safe abortion policies within the reproductive health package of the ministry of health is of utmost importance. The stigma and restrictive legal status around induced abortions in Cameroon mandate a heightened level of clinical suspicion among sick women of reproductive age when received in health facilities, because they might not generally present with classical signs and symptoms of incomplete abortions, and might fail to disclose any induced abortion attempts to hospital staff. Abortion related stigma could scare women from seeking safe abortion services [35]. Cultural and health care facility factors could indirectly perpetuate stigma regarding safe abortions [36]. Abortion related stigma has the potential to hinder access to safe abortion services allowed by the law. Coupled with ignorance regarding legally acceptable circumstances to seek for abortions by women, and at times even health care providers, cases have been reported not to have sought safe (legal) abortion care from pregnancies resulting from rape, sexual coercion and incest [27]. Advocacy efforts in the direction of making the government to recognizing safe abortion care requests as part of the reproductive rights of women must continue. It is idle, hypocritical and unhelpful, to continue to restrict these services while women in need continue to acquire such services “in the shadows” which at times cost them their lives. Implementing currently available, cheap and effective evidence based practice guidelines are possible in Cameroon. The restrictive legal atmosphere does not, and should not justify most of the abortion related complications and deaths recorded in the country.

## Conclusions

Manual Vacuum Aspiration (MVA) kits and misoprostol for post-abortion care should be made widely available. An abortion in most areas of Cameroon is associated with shame and social rejection. Added to this, the restrictive law can prevent women from disclosing abortion attempts or incomplete abortions to health care staff [39, 40]. Liberalization of the law on induced abortions could reduce the number of unsafe abortions. Training health care staff on clinical suspicion of abortions in form of refresher courses or charts could be welcome. This could go a long way towards averting deaths from women, who though seriously ill, might refuse to declare having carried out an abortion. Data presenting the exact distribution and magnitude of unsafe abortions in Cameroon is almost inexistent. Strengthening the Maternal Death Surveillance and Response (MDSR) strategy, as proposed by the WHO and partners could be a unique opportunity to properly ascertain the magnitude and distribution of unsafe abortions, and adequately inform policy makers for timely and region specific interventions to be implemented [24]. Interventions to evaluate and reduce abortion related stigma should also be carefully considered.

## Abbreviations

HIV: Human immunodeficiency virus
MDSR: Maternal death surveillance and response
MVA: Manual vacuum aspiration
PAC: Post - abortion care
WHO: World Health Organization
